# Association Between Metabolic Syndrome and Incident Cervical Cancer: A Retrospective Cohort Study

**DOI:** 10.1155/jobe/3691654

**Published:** 2025-09-29

**Authors:** Linjun Jiang, Akira Okada, Risa Ishida, Hideo Yasunaga

**Affiliations:** ^1^Department of Clinical Epidemiology and Health Economics, School of Public Health, University of Tokyo, Tokyo, Japan; ^2^Department of Prevention of Diabetes and Lifestyle-Related Diseases, Graduate School of Medicine, University of Tokyo, Tokyo, Japan; ^3^Department of Obstetrics and Gynecology, Graduate School of Medicine, The University of Tokyo, Tokyo, Japan

## Abstract

**Objective:**

To determine whether metabolic syndrome is associated with an elevated risk of cervical cancer.

**Methods:**

We retrospectively analyzed data on 1,410,650 women without a history of cancer, using the JMDC Claims Database, a nationwide epidemiological database in Japan, between 2005 and 2022. The look-back period was set at 2 years. Cox regression analyses were conducted to assess cervical cancer risk associated with metabolic syndrome and its components (waist circumference, blood pressure, triglycerides, high-density lipoprotein cholesterol, and fasting plasma glucose). Further, we conducted age-stratified analyses.

**Results:**

Metabolic syndrome was diagnosed in 43,029 participants (median age: 53 years), and 1579 cervical cancer cases were recorded over a median follow-up of 942 days. Multivariable Cox regression analyses showed that metabolic syndrome was associated with a higher cervical cancer incidence (hazard ratio [HR], 1.38; 95% confidence interval [CI], 1.04–1.82). Among the metabolic factors, cancer risk was associated with higher plasma glucose (per 10 mg/dL increase) (HR, 1.04; 95% CI, 1.01–1.08) and lower high-density lipoprotein cholesterol levels (per 10 mg/dL decrease) (HR, 1.06; 95% CI, 1.02–1.10), whereas waist circumference, blood pressure, or triglyceride levels showed no significant relationship. Metabolic syndrome was associated with an increased risk of cervical cancer, with a stronger association observed in younger women in age-stratified analyses (*p* for interaction = 0.004).

**Conclusion:**

Metabolic syndrome was associated with an increased risk of cervical cancer, with a stronger association observed among younger women. Elevated plasma glucose and low high-density lipoprotein cholesterol levels were identified as significant contributing factors.

## 1. Introduction

Cervical cancer is the fourth leading cause of cancer diagnoses and deaths among women globally [1]. The rising incidence of cervical cancer among young women in recent years poses a major concern for global public health [2]. Cervical cancer is predominantly caused by the human papillomavirus (HPV) infection [3], with additional risk factors comprising smoking [4], use of hormonal contraceptives [5], and a history of multiple childbirths [6]. Few studies have examined the link between cervical cancer and non-HPV factors such as metabolic syndrome (MetS), which is an established risk factor for endometrial, colorectal, gastric, and breast malignancies [7–10].

Limited evidence exists on the association between MetS or its components and cervical cancer [10, 11]. Previous studies have discovered that a higher MetS score [12], elevated blood pressure and triglyceride levels [13, 14], and type 2 diabetes [15] were associated with an increased risk of cervical cancer. However, individual metabolic factors show inconsistent association with cervical cancer, and studies on established cervical cancer risk factors remain limited. Furthermore, since cervical cancer often occurs in relatively younger women, research on age-specific associations between MetS and cervical cancer is warranted.

This study aimed to determine whether MetS and its components are associated with an increased cervical cancer risk, adjusting for other risk factors. In addition, we examined the impact of age on the association between MetS and cervical cancer risk.

## 2. Materials and Methods

### 2.1. Study Population

**Figure 1:**
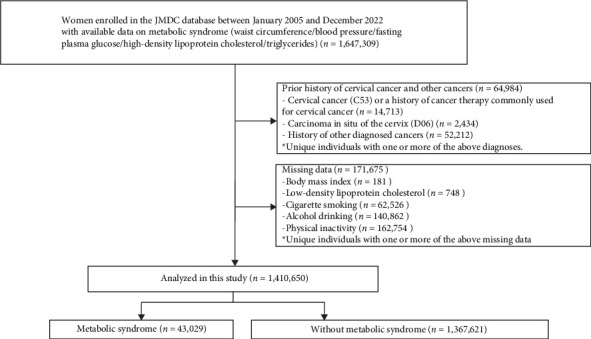
Flowchart for the study population.

We performed a retrospective observational study analyzing data from the JMDC Claims Database, provided by JMDC Inc. in Tokyo, Japan, between January 2005 and December 2022, utilizing health examination records and medical claims information. This comprehensive database encompasses anonymized medical claims and annual health checkup data from over 60 health insurance providers [16–18]. In this study, we included participants whose records contained complete information on MetS components (*n* = 1,647,309) and excluded those with a history of cervical cancer (International Classification of Codes [ICD]-10: C53), prior cancer therapies relevant to cervical cancer (World Health Organization Anatomical Therapeutic Chemical Classification System [WHO-ATC] codes L01XA01, L01XA02, L01XA03, and L01CD01) (*n* = 14,713), carcinoma in situ of the cervix (ICD-10: D06) (*n* = 2434), and those with a history of other diagnosed cancers (*n* = 52,212) who underwent a health checkup within 2 years after insurance enrollment (2-year look-back period). Furthermore, we excluded those with incomplete data for the following key variables: body mass index (BMI) (*n* = 181), low-density lipoprotein cholesterol (*n* = 748), cigarette smoking (*n* = 62,526), alcohol drinking (*n* = 140,862), and physical inactivity (*n* = 162,754). To confirm that excluding participants with missing data did not introduce selection bias, we compared baseline characteristics between the included cohort and those excluded due to missing data (Table S1). The differences in variable distributions were minimal, with all absolute standardized mean differences being < 0.10, indicating no apparent bias introduced by this exclusion process. After applying all exclusion criteria, our final analysis included 1,410,650 participants. Following the application of all exclusion criteria, our final analysis included data from 1,410,650 participants.  Figure 1 shows the flowchart outlining the used data.

### 2.2. Ethics

This study was conducted in accordance with the ethical standards stipulated in the Declaration of Helsinki and was approved by the Ethics Committee of the University of Tokyo (reference number 2018-10862). As all data within this dataset were anonymized, the Ethics Committee waived the necessity for obtaining informed consent. All data conformed to the guidelines established by the International Conference on Harmonization [19].

### 2.3. MetS Definition

We collected medical examination data using standardized procedures, which included the measurements of BMI, waist circumference, fasting plasma glucose level, dyslipidemia, blood pressure, history of hypertension and diabetes mellitus, and fasting laboratory values. MetS was diagnosed based on Japanese criteria [20], requiring an increased waist circumference (≥ 90 cm at the umbilical level for women) and at least two of the following conditions: impaired fasting glucose/diabetes (fasting plasma glucose level ≥ 110 mg/dL or use of glucose-lowering medications), elevated blood pressure/hypertension (systolic blood pressure ≥ 130 mmHg or diastolic blood pressure ≥ 85 mmHg), and dyslipidemia (triglyceride level ≥ 150 mg/dL, high-density lipoprotein [HDL] cholesterol level of < 40 mg/dL, or use of lipid-lowering medications).

### 2.4. Other Covariates

We collected additional data on smoking habits (current smokers) and alcohol use (daily drinkers) through questionnaires. Insufficient physical activity was defined as exercising less than twice a week for at least 30 min, based on a health checkup questionnaire as previously described [7]. We defined obesity as BMI of ≥ 25 kg/m^2^.

### 2.5. Outcome

The outcome of this research was a cervical cancer diagnosis, identified using the ICD-10 code C53.

### 2.6. Statistical Analysis

Continuous variables were evaluated using unpaired *t*-tests, while categorical variables were examined using chi-square tests. The cumulative incidence of cervical cancer events was compared between patients with and without MetS using Kaplan–Meier curves and log-rank tests. We employed Cox proportional hazards models to assess the association between MetS and the risk of developing cervical cancer, as well as to examine the relationship between each MetS criterion component and incident cervical cancer cases.

Model 1 included only MetS (unadjusted model), and Model 2 (adjusted model) included MetS, age, BMI, low-density lipoprotein cholesterol level, alcohol drinking frequency, current smoking status, and physical inactivity. When a significant association was found between any MetS component and cancer risk, we further assessed whether the interaction term was significantly correlated with the risk. We incorporated the term into the fully adjusted model when the interaction term reached statistical significance. Furthermore, we generated quintile-based Kaplan–Meier curves for significant MetS components to assess potential dose–response relationships with cervical cancer incidence.

An age-stratified analysis was conducted using the categories of < 40, 40–59, and ≥ 60 years, corresponding to the reproductive, perimenopausal, and postmenopausal stages of a woman's life, which are relevant to both metabolic risk and cervical carcinogenesis [21]. *p* values for interactions between these age groups were calculated to assess any significant age-related modification effects.

Six sensitivity analyses were performed. The first two focused on the relationship between MetS and incident cervical cancer using the International Diabetes Federation (IDF) criteria for Asians and the National Cholesterol Education Program Adult Treatment Panel III (NCEP/ATP III) criteria. Third, we defined the endpoint for cervical cancer events as the first occurrence of either cervical cancer-related surgery or anticancer drug prescription. Fourth, for women aged 18–39, we used HPV vaccination data from the Japanese Ministry of Health, Labour and Welfare. Women born between 1994 and 1999 were categorized as the “vaccinated generation,” while others were classified as the “unvaccinated generation” [22]. Generation status was included as a potential confounder in the Cox regression model. Fifth, we conducted multiple imputation for participants with missing values for BMI (*n* = 181), low-density lipoprotein cholesterol (*n* = 748), smoking status (*n* = 62,526), alcohol consumption (*n* = 140,862), and physical inactivity (*n* = 162,754). Multiple imputation using chained equations was conducted for 20 iterations to handle missing data under the assumption that the data were missing at random. Hazard ratios (HRs) with corresponding standard errors were estimated using Rubin's rules. Sixth, we applied a Fine-Gray subdistribution hazard model in a subgroup analysis limited to women aged ≥ 60 years to account for competing risks of death, treating cervical cancer as the event of interest and death as a competing event.

The IDF defines MetS for Asian populations as having an increased waist circumference (≥ 80 cm for women), along with two or three of the following conditions: impaired fasting glucose/diabetes (fasting plasma glucose ≥ 100 mg/dL or use of glucose-lowering medications), elevated blood pressure/hypertension (systolic blood pressure ≥ 130 mmHg, diastolic blood pressure ≥ 85 mmHg, or use of antihypertensive medications), and dyslipidemia (for women, triglycerides ≥ 150 mg/dL or HDL cholesterol < 50 mg/dL, or use of lipid-lowering medications) [23]. The NCEP/ATP III defines elevated blood pressure/hypertension, impaired fasting glucose/diabetes, and dyslipidemia similarly; MetS is diagnosed using any three or four of the following: elevated blood pressure/hypertension, impaired fasting glucose/diabetes, dyslipidemia, or an increased waist circumference (≥ 88 cm for women) [24].

## 3. Results

The clinical characteristics of the study population (*n* = 1,410,650) are presented in Table 1. The median age (interquartile range) of the participants was 44 years (37–52), and MetS was observed in 43,029 participants (3.05%). Participants with MetS were older, more likely to be obese, and current smokers compared with those without MetS (Table 1).

**Figure 2:**
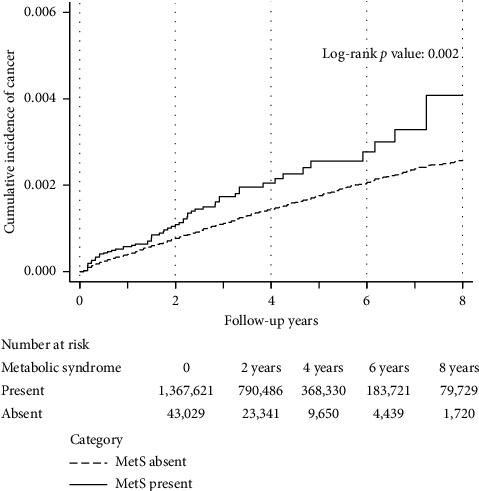
Kaplan–Meier curves for cervical cancer.

During the median follow-up of 942 days (interquartile range: 425–1552), 1579 cases of cervical cancer were recorded. Kaplan–Meier curves demonstrated a significant difference in cumulative cervical cancer incidence between patients with and without MetS (Figure 2). The incidence rates for cervical cancer were higher in patients with MetS (5.28 [95% confidence interval (CI), 4.13–6.75] per 10,000 person-years) than in those without MetS (3.54 [3.37–3.72] per 10,000 person-years), corresponding to an absolute risk difference of 1.74 (95% CI, 0.43–3.05) per 10,000 person-years. Univariate Cox regression analysis (Model 1) showed that MetS prevalence was associated with a higher risk of developing cervical cancer (HR, 1.47; 95% CI, 1.15–1.89). Multivariable Cox regression analysis (Model 2) demonstrated that MetS was associated with greater cervical cancer risk (HR, 1.38; 95% CI, 1.04–1.82) (Table 2). Among metabolic factors, higher plasma glucose (per 10 mg/dL increase; HR, 1.04; 95% CI, 1.01–1.08) and lower HDL cholesterol (per 10 mg/dL decrease; HR, 1.06; 95% CI, 1.02–1.10) levels were associated with an increased cervical cancer risk, whereas waist circumference, blood pressure, and triglyceride levels were not significantly associated with the risk (Table 3). Incorporating the interaction term between plasma glucose and HDL cholesterol levels yielded an HR of 1.00 (95% CI, 0.98–1.02; *p* for interaction = 0.81), indicating no significant multiplicative interaction. These findings suggest that higher plasma glucose and lower HDL cholesterol levels independently contribute to increased cervical cancer risk. When stratified by quintiles of metabolic components, both fasting plasma glucose (*p*=0.002) and HDL cholesterol (*p*=0.001, Figure S1) levels showed significant differences in cumulative cervical cancer incidence across quintiles, although neither demonstrated a strictly monotonic trend.

**Figure 3:**
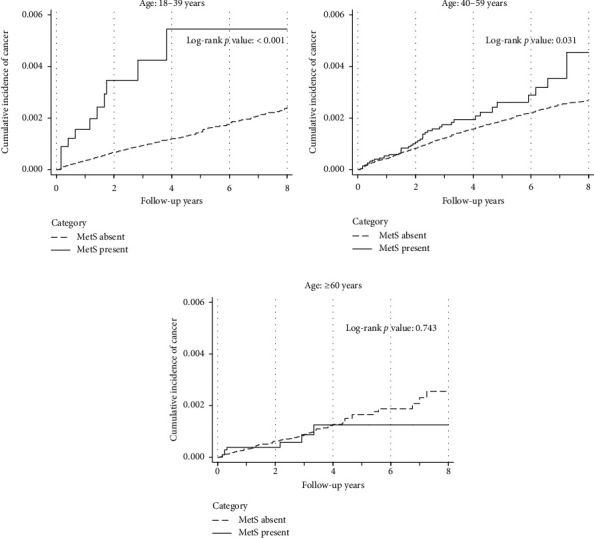
Kaplan–Meier survival curves stratified by age group (< 40, 40–59, and ≥ 60 years).

In the age-stratified analysis, 432,789 (30.68%), 847,041 (60.05%), and 130,811 (9.27%) participants were aged 18–39 years, 40–59 years, and ≥ 60 years, respectively. The association between MetS and cervical cancer was examined within each age group. Unadjusted models showed that MetS was associated with cervical cancer in both the 18–39 years (HR, 3.78; 95% CI, 2.08–6.89) and 40–59 years age (HR, 1.39; 95% CI, 1.03–1.87) groups. Multivariable Cox regression analyses demonstrated that MetS was significantly associated with a higher risk of incident cervical cancer in both the 18–39 years (HR, 2.73; 95% CI, 1.38–5.39) and 40–59 years age groups (HR, 1.58; 95% CI, 1.13–2.20, *p* for interaction = 0.004). However, in the ≥ 60 years age group, MetS was not associated with a higher risk of cervical cancer in either the unadjusted (HR, 0.88; 95% CI, 0.41–1.90) or adjusted models (HR, 0.58; 95% CI, 0.24–1.37) (Table 2). Kaplan–Meier analysis confirmed these associations, showing significantly higher cumulative cervical cancer incidence in MetS-positive women aged 18–39 (*p* < 0.001) and 40–59 (*p*=0.031) years, but not in those aged ≥ 60 years (*p*=0.743) (Figure 3). Analyses of individual components of MetS showed that, in the 18–39 years age group, only higher fasting plasma glucose level was significantly associated with incident cervical cancer (HR, 1.08; 95% CI, 1.01–1.15). In the 40–59 years age group, both higher fasting plasma glucose (HR, 1.05; 95% CI, 1.01–1.09) and lower HDL cholesterol levels (HR, 1.06, 95% CI, 1.01–1.10) were associated with incident cervical cancer. However, in the ≥ 60 years age group, none of the metabolic risk factors showed a significant association with incident cervical cancer (Table 3).

Table 4 shows the results of the six sensitivity analyses. The first and second sensitivity analyses used different definitions of MetS, yielding results similar to those of the main analysis. When using the IDF criteria, MetS was associated with a higher cervical cancer incidence (HR, 1.40; 95% CI, 1.17–1.68). Using the NCEP/ATP III criteria, similar results were obtained (HR, 1.35; 95% CI, 1.09–1.66). MetS was associated with an increased risk of cervical cancer events in the third (HR, 1.41; 95% CI, 1.08–1.84) and fourth sensitivity analysis (HR, 2.74; 95% CI, 1.39–5.41). The fifth sensitivity analysis using multiple imputation to account for missing data yielded results consistent with those of the primary analysis (HR, 1.41; 95% CI, 1.09–1.83). In the sixth sensitivity analysis using a Fine-Gray competing risk model among women aged ≥ 60 years, the association remained nonsignificant (subdistribution HR, 0.58; 95% CI, 0.22–1.48), supporting the age-stratified findings from the main analysis.

## 4. Discussion

In this analysis of 1,410,650 women aged 18–74 years, using a nationwide health checkup and insurance claims dataset, we found that MetS was associated with elevated cervical cancer incidence after adjusting for other known risk factors. This association was consistent when using diagnostic criteria for MetS from outside of Japan. Notably, the association between MetS and cervical cancer risk was stronger in younger women. In addition, elevated fasting plasma glucose and reduced HDL cholesterol levels were independently associated with increased cervical cancer risk.

The positive association between MetS and cervical cancer observed in this study aligns with those of previous studies. European cohorts have consistently demonstrated a positive association between elevated metabolic risk scores and increased cervical cancer risk [10–12]. A previous study in the United States, using data from the National Health and Nutrition Examination Survey, reported that the odds ratio of MetS was 1.82 for a history of cervical cancer [11]. Although these studies indicated a potential association, studies adjusting established risk factors have been limited. Our study expanded on this by adjusting for smoking history, alcohol consumption status, and physical inactivity. Even after adjusting for these factors, MetS remained significantly associated with increased cervical cancer risk, and this association was consistent across Japanese, IDF, and NCEP/ATP III criteria, highlighting the robustness of our findings.

We found that age modified the relationship between MetS and cervical cancer incidence. In our study, age-stratified analyses revealed a higher incidence of cervical cancer and a positive association between MetS and cervical cancer risk in younger Japanese women. Our findings contrast with those of a previous European study [12], which reported a lower overall cervical cancer incidence and found that MetS was associated with cancer risk primarily in older women. We also confirmed that the absence of association between MetS and cervical cancer in women aged ≥ 60 years was not due to competing mortality risks, as the Fine-Gray competing-risks analysis yielded results consistent with our primary analysis. This discrepancy may be due to population-specific factors, including ethnic and genetic variations, lifestyle differences, and varying prevalence of MetS components across different age groups and populations.

Our analysis of individual metabolic factors and cervical cancer risk revealed several key insights. In the overall analysis, we found that elevated fasting plasma glucose and low HDL cholesterol levels were associated with an increased cervical cancer risk, while triglyceride levels, blood pressure, and obesity were not correlated with the risk. Age-stratified analyses indicated that the association between fasting plasma glucose levels and cancer risk remained significant only in women aged 18–39 and 40–59 years, while that between HDL cholesterol levels and cancer risk remained significant only in women aged 18–39 years. The association with glucose was consistent with those of previous findings, particularly in younger and middle-aged women [12]. This association remained significant after adjusting for other metabolic factors, suggesting a potential mechanism involving hyperglycemia-induced insulin resistance promoting cancer cell proliferation and metastasis [25, 26]. The relationship with HDL cholesterol differed from those of a prior study [11]. The absence of an association with triglycerides and blood pressure contrasted with those of some earlier studies [13, 14]. Although these factors may potentially contribute to cancer progression through oxidative stress and inflammation [27, 28], we found no relationship between obesity (defined by BMI of ≥ 25 kg/m^2^) and cervical cancer risk, contrary to previous research [12].

Emerging evidence suggests that obesity-related biological mechanisms contribute to cervical carcinogenesis beyond the associations captured by anthropometric measures alone. Recent evidence suggests that dysfunction of adipose tissue, particularly visceral fat, contributes to cancer development through mechanisms not fully reflected by anthropometric measures [29, 30]. In obesity, adipose tissue functions as an active endocrine and immune organ, releasing adipokines and proinflammatory cytokines, including tumor necrosis factor-alpha and interleukin-6, and attracting immune cells such as macrophages [31]. Once free fatty acids and other signals activate these macrophages, they further stimulate pathways such as nuclear factor κappa B and amplify the secretion of proinflammatory mediators [31–33]. The resulting local and systemic inflammation creates a protumorigenic environment that supports cellular proliferation, angiogenesis, and invasion, potentially increasing cervical cancer risk independent of BMI [32, 33]. This discrepancy between our findings and those of some previous studies may be attributed to variations in study populations, differences in lifestyles or environmental exposures, or adjustments for other confounders. Further studies should explore the metabolic mechanisms underlying cervical cancer risk.

Our study has several strengths and important clinical implications. A major strength is the implementation of a large nationwide longitudinal health checkup database with high participant engagement and outcome identification rates, further enhanced by electronic linkages to health insurance claim records. The key message of our study is that assessing MetS could help identify younger individuals at a higher risk for cervical cancer, which is increasingly affecting younger women globally [2]. This finding underscores the need for general physicians and endocrinologists treating patients with MetS to be aware of the potential increased risk of cervical cancer, especially in younger women. In addition, our study's identification of specific metabolic factors, such as high fasting plasma glucose and low HDL cholesterol level, as being particularly associated with increased risk of developing cervical cancer suggests that targeted management of these factors might be beneficial.

This study had some limitations. First, our multivariable analyses lacked detailed information on potential confounding factors, such as socioeconomic status, sexual behavior, HPV infection status, and reproductive factors, which might have influenced the association between MetS and cervical cancer incidence. In addition, HPV vaccination status was estimated based on general Japanese vaccination trends, lacking specific individual-level data. Finally, the median follow-up period of 2.6 years in our study was relatively short given the typically long-term development of cervical cancer (10–20 years) [34]. Therefore, our results primarily reflect short- to mid-term risks and may not capture the full long-term effects of metabolic factors.

## 5. Conclusion

In conclusion, this large-scale study demonstrates a significant association between MetS and increased cervical cancer risk, especially among younger women. The identification of high fasting plasma glucose and low HDL cholesterol levels as key risk factors suggests that assessing MetS could be valuable in identifying individuals at a higher risk for cervical cancer, enabling earlier intervention.

## Figures and Tables

**Table 1:** Characteristics of the study participants.

	Metabolic syndrome		*p* value
	Absent (*N* = 1,367,621)	Present (*N* = 43,029)	
Body mass index (kg/m^2^)	21 (19.2–23.3)	29.4 (27.1–32.4)	< 0.001
Waist circumference (cm)	75.5 (70–82)	97 (93–103)	< 0.001
Age (years)	44 (37–52)	53 (47–60)	< 0.001
Systolic blood pressure (mmHg)	112 (102–123)	136 (128–146)	< 0.001
Diastolic blood pressure (mmHg)	69 (62–77)	83 (76–90)	< 0.001
Fasting plasma glucose (mg/dL)	89 (84–95)	110 (96–123)	< 0.001
Triglycerides (mg/dL)	64 (48–89)	146 (98–189)	< 0.001
High-density lipoprotein cholesterol (mg/dL)	71 (61–82)	56 (48–65)	< 0.001
Low-density lipoprotein cholesterol (mg/dL)	114 (95–136)	131 (110–155)	< 0.001
Obesity	198,692 (14.5%)	39,781 (92.5%)	< 0.001
Current smoker	147,473 (10.8%)	6036 (14.0%)	< 0.001
Drinking frequency			
Regularly	171,877 (12.6%)	4082 (9.5%)	< 0.001
Occasionally	455,303 (33.3%)	11,485 (26.7%)	
Rarely/never	740,441 (54.1%)	27,462 (63.8%)	
Regular exercise habits	232,225 (17.0%)	7326 (17.0%)	0.80
Increased waist circumference	109,466 (8.0%)	43,029 (100.0%)	< 0.001
Elevated blood pressure/hypertension	260,474 (19.0%)	38,361 (89.2%)	< 0.001
Impaired fasting glucose/diabetes	47,431 (3.5%)	23,585 (54.8%)	< 0.001
Dyslipidemia	131,503 (9.6%)	34,718 (80.7%)	< 0.001

*Note:* The following definitions were used: obesity as body mass index ≥ 25 kg/m^2^, increased waist circumference as ≥ 90 cm at the umbilical level, elevated blood pressure or hypertension as systolic blood pressure ≥ 130 mmHg or diastolic blood pressure ≥ 85 mmHg, and impaired fasting glucose or diabetes as fasting plasma glucose ≥ 110 mg/dL or use of glucose-lowering medications. Data are expressed as median (interquartile range) or number (percentage).

**Table 2:** The frequency of events, corresponding incidence rates, and hazard ratios for cervical cancer events in participants with and without metabolic syndrome, stratified by the age groups.

Population	Metabolic syndrome	Number	No. of events	Incidence rate (95% CI), per 10,000 person-years		Model 1 (unadjusted), HR (95% CI)		Model 2 (adjusted), HR (95% CI)	
Overall	Absent	1,367,621	1515	3.54	(3.37–3.72)				
	Present	43,029	64	5.28	(4.13–6.75)	1.47	(1.15–1.89)	1.38	(1.04–1.82)

Age 18–39 years	Absent	429,379	375	3.09	(2.79–3.42)				
	Present	3419	11	11.70	(6.48–21.12)	3.78	(2.08–6.89)	2.73	(1.38–5.39)

Age 40–59 years	Absent	818,539	1050	3.79	(3.57–4.02)				
	Present	28,502	46	5.35	(4.01–7.14)	1.39	(1.03–1.87)	1.58	(1.13–2.20)

Age ≥ 60 years	Absent	119,703	90	3.09	(2.51–3.80)				
	Present	11,108	7	2.71	(1.29–5.69)	0.88	(0.41–1.90)	0.58	(0.24–1.37)

*Note:* Model 2 includes adjustment for age, BMI, low-density lipoprotein cholesterol, cigarette smoking, alcohol drinking, and physical inactivity.

Abbreviations: CI = confidence interval; HR = hazard ratio.

**Table 3:** Hazard ratios (95% confidence intervals) of each metabolic risk factor for incident cervical cancer among the overall and age-stratified populations.

Variable	Overall population		Age 18–39 years		Age 40–59 years		Age ≥ 60 years	
Waist circumference (per 10 cm increase)	0.98	(0.88–1.09)	1.00	(0.80–1.26)	0.95	(0.84–1.08)	1.15	(0.76–1.73)
Mean blood pressure (per 10 mmHg increase)	0.99	(0.94–1.03)	0.96	(0.86–1.06)	1.01	(0.96–1.06)	0.97	(0.82–1.15)
Fasting plasma glucose (per 10 mg/dL increase)	1.04	(1.01–1.08)	1.08	(1.01–1.15)	1.05	(1.01–1.09)	0.94	(0.82–1.07)
Triglycerides (per 10 mg/dL increase)	1.01	(1.00–1.02)	1.02	(1.00–1.03)	1.00	(0.99–1.02)	1.01	(0.98–1.04)
High-density lipoprotein cholesterol (per 10 mg/dL decrease)	1.06	(1.02–1.10)	1.06	(0.98–1.15)	1.06	(1.01–1.10)	1.09	(0.95–1.26)

*Note:* Adjusted for age, low-density lipoprotein cholesterol level, cigarette smoking, alcohol consumption, and physical inactivity.

**Table 4:** Frequency of events, corresponding incidence rates, and hazard ratios for cervical cancer events among participants by metabolic syndrome in six sensitivity analyses.

	Metabolic syndrome	Number	No. of events	Incidence rate (95% CI), per 10,000 person-years		Model 1 (unadjusted), HR (95% CI)		Model 2 (adjusted), HR (95% CI)	
Sensitivity analysis 1 (metabolic syndrome defined by IDF criteria)	Absent	1,283,863	1398	3.47	(3.29–3.65)				
	Present	126,787	181	4.96	(4.29–5.74)	1.41	(1.21–1.65)	1.40	(1.17–1.68)

Sensitivity analysis 2 (metabolic syndrome defined by NCEP/ATP III criteria)	Absent	1,318,056	1449	3.50	(3.33–3.69)				
	Present	92,594	130	4.94	(4.16–5.87)	1.39	(1.16–1.67)	1.35	(1.09–1.66)

Sensitivity analysis 3 (defining outcome as surgery or chemotherapy)	Absent	1,367,621	1114	2.60	(2.46–2.76)				
	Present	43,029	58	4.79	(3.70–6.20)	1.8	(1.38–2.34)	1.47	(1.09–1.99)

Sensitivity analysis 4 (adding adjustment for HPV vaccination status in participants aged 18–39 years)	Absent	429,379	375	3.09	(2.79–3.42)				
	Present	3419	11	11.7	(6.48–21.12)	3.63	(1.99–6.60)	2.74	(1.39–5.41)

Sensitivity analysis 5 (multiple imputation)	Absent	1,532,241	1748	3.58	(3.42–3.76)				
	Present	48,261	75	5.44	(4.34–6.82)	1.50	(1.19–1.89)	1.38	(1.04–1.82)

Sensitivity analysis 6 (Fine-Gray competing risk analysis in women aged ≥ 60 years)	Absent	119,703	90	3.09	(2.51–3.80)				
	Present	11,108	7	2.71	(1.29–5.69)	0.88	(0.41–1.90)	0.58	(0.22–1.48)

*Note:* Adjusted estimates were adjusted for age, low-density lipoprotein cholesterol level, cigarette smoking, alcohol consumption, and physical inactivity.

Abbreviations: CI = confidence interval, HPV = human papillomavirus, HR = hazard ratio, IDF = International Diabetes Federation, and NCEP/ATP III = National Cholesterol Education Program Adult Treatment Panel III.

## Data Availability

Data used in this study are from JMDC's health insurance database. The data are available for surveys, research, and commercial use on a fee basis. Potential users can obtain access through JMDC's website (https://www.jmdc.co.jp/en/bigdata) after completing the contract for use of specific data.

## Supporting Information

Table S1: baseline characteristics of the included and excluded participants.

Figure S1: Kaplan–Meier curves stratified by quintiles of fasting plasma glucose and high-density lipoprotein cholesterol, based on the presence of metabolic syndrome.

## References

[1] Siegel R. L., Giaquinto A. N., Jemal A. (2024). Cancer Statistics, 2024. *CA: A Cancer Journal for Clinicians*.

[2] Bray F., Laversanne M., Sung H. (2024). Global Cancer Statistics 2022: GLOBOCAN Estimates of Incidence and Mortality Worldwide for 36 Cancers in 185 Countries. *CA: A Cancer Journal for Clinicians*.

[3] Schiffman M. H., Castle P. (2003). Epidemiologic Studies of a Necessary Causal Risk Factor: Human Papillomavirus Infection and Cervical Neoplasia. *Journal of the National Cancer Institute*.

[4] Beral V., Berrington de González A., Colin D. (2006). Carcinoma of the Cervix and Tobacco Smoking: Collaborative Reanalysis of Individual Data on 13,541 Women With Carcinoma of the Cervix and 23,017 Women Without Carcinoma of the Cervix From 23 Epidemiological Studies. *International Journal of Cancer*.

[5] Smith J. S., Green J., de Gonzalez A. B. (2003). Cervical Cancer and Use of Hormonal Contraceptives: A Systematic Review. *The Lancet*.

[6] O’Brien K. M., Weinberg C. R., D’Aloisio A. A., Moore K. R., Sandler D. P. (2021). The Association Between Douching, Genital Talc Use, and the Risk of Prevalent and Incident Cervical Cancer. *Scientific Reports*.

[7] Jimba T., Kaneko H., Yano Y. (2021). Relation of the Metabolic Syndrome to Incident Colorectal Cancer in Young Adults Aged 20 to 49 Years. *The American Journal of Cardiology*.

[8] Berrino F., Villarini A., Traina A. (2024). Metabolic Syndrome and Breast Cancer Prognosis. *Breast Cancer Research and Treatment*.

[9] Ni J., Zhu T., Zhao L. (2015). Metabolic Syndrome Is an Independent Prognostic Factor for Endometrial Adenocarcinoma. *Clinical and Translational Oncology*.

[10] Stocks T., Bjørge T., Ulmer H. (2015). Metabolic Risk Score and Cancer Risk: Pooled Analysis of Seven Cohorts. *International Journal of Epidemiology*.

[11] Penaranda E. K., Shokar N., Ortiz M. (2013). Relationship Between Metabolic Syndrome and History of Cervical Cancer Among a US National Population. *ISRN Oncology*.

[12] Ulmer H., Bjørge T., Concin H. (2012). Metabolic Risk Factors and Cervical Cancer in the Metabolic Syndrome and Cancer Project (Me-Can). *Gynecologic Oncology*.

[13] Shen T., Zhao J., Li W. (2023). Hypertension and Hyperglycaemia Are Positively Correlated With Local Invasion of Early Cervical Cancer. *Frontiers in Endocrinology*.

[14] Ulmer H., Borena W., Rapp K. (2009). Serum Triglyceride Concentrations and Cancer Risk in a Large Cohort Study in Austria. *British Journal of Cancer*.

[15] Yuan S., Kar S., Carter P. (2020). Is Type 2 Diabetes Causally Associated With Cancer Risk? Evidence From a Two-Sample Mendelian Randomization Study. *Diabetes*.

[16] Ueno K., Kaneko H., Suzuki Y. (2024). Metabolic Syndrome and Cardiovascular Disease in Cancer Survivors. *Journal of Cachexia, Sarcopenia and Muscle*.

[17] Itoh H., Kaneko H., Kiriyama H. (2021). Metabolically Healthy Obesity and the Risk of Cardiovascular Disease in the General Population: Analysis of a Nationwide Epidemiological Database. *Circulation Journal*.

[18] Kaneko H., Yano Y., Itoh H. (2021). Untreated Hypertension and Subsequent Incidence of Colorectal Cancer: Analysis of a Nationwide Epidemiological Database. *Journal of the American Heart Association*.

[19] Dixon J. R. (1999). The International Conference on Harmonization Good Clinical Practice Guideline. *Quality Assurance*.

[20] Matsuzawa Y. (2005). Metabolic Syndrome—Definition and Diagnostic Criteria in Japan. *Journal of Atherosclerosis and Thrombosis*.

[21] Harlow S. D., Derby C. A. (2015). Women’s Midlife Health: Why the Midlife Matters. *Women’s Midlife Health*.

[22] Alberti K. G. M. M., Zimmet P., Shaw J. (2006). Metabolic Syndrome—A New World-Wide Definition. A Consensus Statement From the International Diabetes Federation. *Diabetic Medicine*.

[23] Grundy S. M., Cleeman J. I., Daniels S. R. (2005). Diagnosis and Management of the Metabolic Syndrome: An American Heart Association/National Heart, Lung, and Blood Institute Scientific Statement. *Circulation*.

[24] Nakagawa S., Ueda Y., Yagi A., Ikeda S., Hiramatsu K., Kimura T. (2020). Corrected Human Papillomavirus Vaccination Rates for Each Birth Fiscal Year in Japan. *Cancer Science*.

[25] Li W., Zhang X., Sang H. (2019). Effects of Hyperglycemia on the Progression of Tumor Diseases. *Journal of Experimental & Clinical Cancer Research*.

[26] Mardilovich K., Pankratz S. L., Shaw L. M. (2009). Expression and Function of the Insulin Receptor Substrate Proteins in Cancer. *Cell Communication and Signaling*.

[27] Egami K., Murohara T., Shimada T. (2003). Role of Host Angiotensin II Type 1 Receptor in Tumor Angiogenesis and Growth. *Journal of Clinical Investigation*.

[28] Hashemzehi M., Beheshti F., Hassanian S. M., Ferns G. A., Khazaei M., Avan A. (2020). Therapeutic Potential of Renin Angiotensin System Inhibitors in Cancer Cells Metastasis. *Pathology, Research & Practice*.

[29] Lacey J. V., Swanson C. A., Brinton L. A. (2003). Obesity as a Potential Risk Factor for Adenocarcinomas and Squamous Cell Carcinomas of the Uterine Cervix. *Cancer*.

[30] Green J., Berrington de Gonzalez A., Sweetland S. (2003). Risk Factors for Adenocarcinoma and Squamous Cell Carcinoma of the Cervix in Women Aged 20–44 Years: The UK National Case-Control Study of Cervical Cancer. *British Journal of Cancer*.

[31] Tilg H., Moschen A. R. (2006). Adipocytokines: Mediators Linking Adipose Tissue, Inflammation and Immunity. *Nature Reviews Immunology*.

[32] Li B., Sun S., Li J. J., Yuan J. P., Sun S. R., Wu Q. (2023). Adipose Tissue Macrophages: Implications for Obesity-Associated Cancer. *Military Medical Research*.

[33] Iyengar N. M., Gucalp A., Dannenberg A. J., Hudis C. A. (2016). Obesity and Cancer Mechanisms: Tumor Microenvironment and Inflammation. *Journal of Clinical Oncology*.

[34] Burger E. A., De Kok I. M. C. M., Groene E. (2020). Estimating the Natural History of Cervical Carcinogenesis Using Simulation Models: A CISNET Comparative Analysis. *Journal of the National Cancer Institute*.

