# Supplementary material for: Association Between Metabolic Syndrome and Incident Cervical Cancer: A Retrospective Cohort Study
Source: J Obes. 2025 Sep 29;2025:3691654. doi: 10.1155/jobe/3691654 (PMC12500346; doi:10.1155/jobe/3691654)

Table S1: Baseline characteristics of included and excluded participants

	Excluded cohort N=171,675	Included cohort N=1,410,650	Absolute standardized difference
Metabolic Syndrome	5,307 (3.1%)	43,029 (3.1%)	0.2%
Waist circumference (cm)	77.0 (10.0)	77.4 (9.9)	3.4%
Age (years)	44.0 (11.0)	44.7 (10.8)	6.6%
Systolic blood pressure (mmHg)	115.0 (16.2)	114.5 (16.6)	2.8%
Diastolic blood pressure (mmHg)	70.2 (11.3)	70.1 (11.4)	0.7%
Fasting plasma glucose (mg/dl)	90.9 (13.8)	91.0 (13.6)	1.4%
Triglycerides (mg/dl)	79.4 (51.5)	78.1 (50.5)	2.6%
High-Density lipoprotein cholesterol (mg/dl)	71.2 (16.2)	71.7 (16.3)	3.0%
Increased waist circumference	18,085 (10.5%)	152,495 (10.8%)	0.9%
Elevated blood pressure/hypertension	36,789 (21.4%)	298,835 (21.2%)	0.6%
Impaired fasting glucose/diabetes	8,980 (5.2%)	71,016 (5.0%)	0.9%
Dyslipidemia	21,200 (12.3%)	166,221 (11.8%)	1.7%

Note: increased waist circumference, ≥90 cm at the umbilical level; and impaired fasting glucose/diabetes, fasting blood glucose level ≥110 mg/dL or use of glucose-lowering medications; and elevated blood pressure/hypertension, systolic blood pressure ≥130 mmHg or diastolic blood pressure ≥85 mmHg.

Data are expressed as mean (standard deviation) or number (percentage).

Figure S1: Kaplan–Meier curves stratified by quintiles of fasting plasma glucose and high-density lipoprotein cholesterol, based on the presence of metabolic syndrome

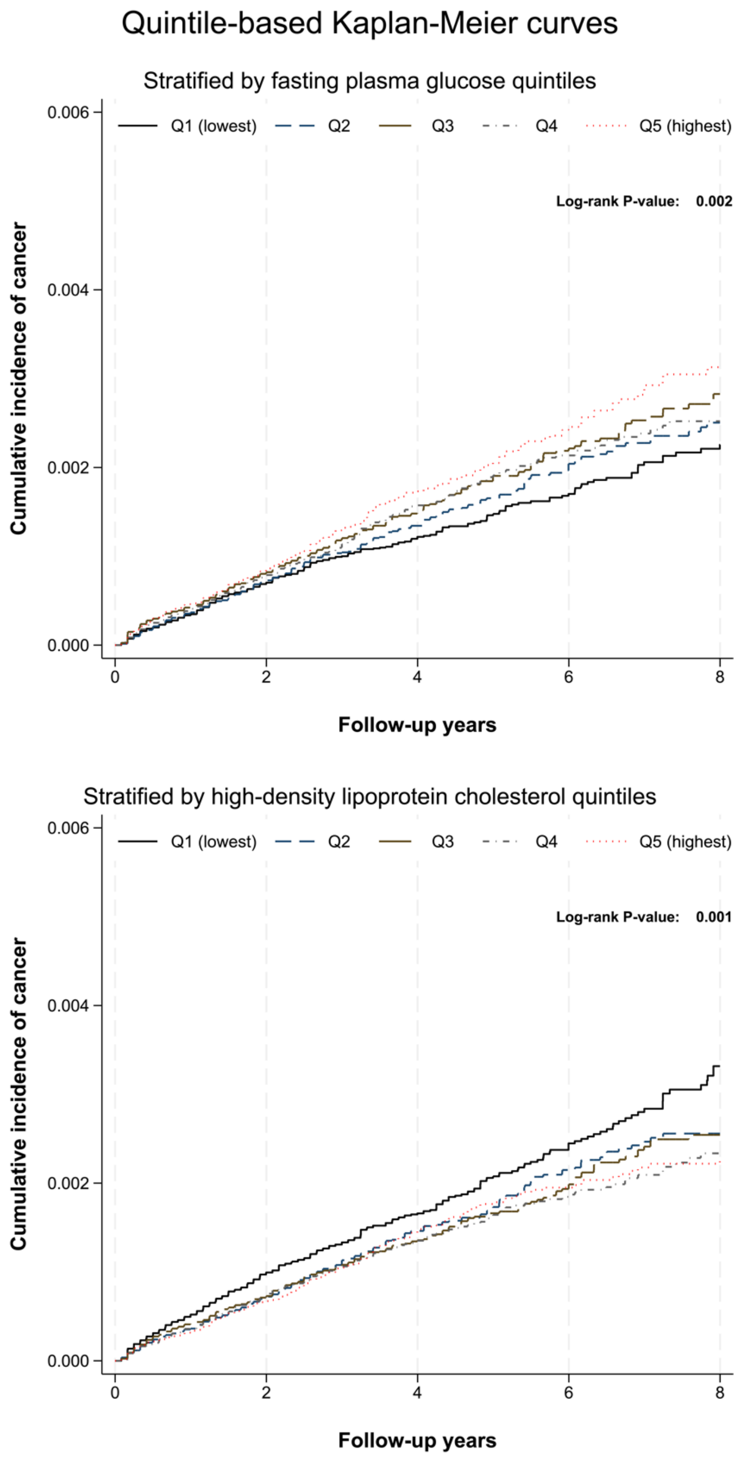

Supplement: Supporting Information — Additional supporting information can be found online in the Supporting Information section. [file 3691654.f1.pdf]
